# Indents

**Published:** 1923-04

**Authors:** 


### INDENTS

**☞Brief Articles of Technical or Scientific Value will receive notice and comment. Contributions invited.**

---

How to Set Up Full Upper and Lower Dentures. It is assumed that the impressions and bite have been taken; the models made and attached to the articulator; baseplates of Dentsply Baseplate Composition shaped over the models, and ridges of Dentsply Baseplate Wax formed of the correct height for the case and of the fullness necessary to proper expression of the lips.

It is assumed also that the location of the median line, the high line and proposed location of the distal proximal surface of the cuspid has been marked on the wax and Trubyte teeth selected of the required form and size.

To avoid too long a presentation and possible confusion, all *esthetic considerations* are here omitted and only the more important *mechanical steps* are described.

The methods described are those followed in the Laboratory of this division.

Illustrations are given in the book Prosthetic Articulation, by George Wood Clapp, D. D. S. Dentists desiring a copy of this book may obtain it for $1.00, either through their dealer or direct from us if remittance accompanies the order.

---

Placing the Upper Incisors. Make sure that the upper wax trial plate rim touches the lower wax trial plate rim firmly in the molar region on both sides. Remove the Incisor Guide Pin.

Cut in the upper ridge, just at one side of the median line, a place large enough and deep enough to permit setting an upper central so that its labial surface is on a line with the labial surface of adjacent wax. Set the tooth so that its incisal edge touches the labial margin of the lower ridge. Wax it firmly in position. If the tooth is too long to set properly, pare the baseplate until the cast can be seen. If this does not give enough room, grind the ridgelap of the tooth.

Set the adjoining lateral in same manner as central but so that its cutting edge is about ½ mm. above the surface of the lower ridge. The lateral may be set irregularly, for artistic effect, but should not be made to lap the central. Wax it firmly.

Cut a place for the upper cuspid and set it so that its tip touches the lower ridge. Rotate it so that the distal portion of the labial surface is nearly parallel with the median line.

If the wax ridge of the upper trial plate was made to restore the desired expression to the lips, the labial inclinations of the teeth will be determined by setting them to the fullness of the wax. The general principal of inclinations is described on the following page.

When the upper incisors of one side have been set, set the upper incisors of the other side in like manner and wax firmly in position.

Replace the Incisor Guide Pin and keep it in place while setting all the biscuspids and molars.

---

Setting the Upper Posteriors. Set the upper biscuspids and molars on both sides. The vertical axes of these teeth should be correctly sloped in two directions. These directions may be described as the mesial and the buccal.

The buccal cusps of each upper first bicuspid should touch the lower wax ridge, but the lingual cusp should not.

Both cusps of the upper second bicuspid should touch the lower wax ridge. This is the only upper tooth of which all the cusps touch the lower wax rim.

The lingual cusps of the upper first molar touch the occlusal surface of the lower ridge, but the buccal cusps are elevated about ¾ mm. above the wax ridge.

The lingual cusps of the upper second molars touch the lower trial plate, but the buccal cusps are elevated, on the average, about 1½ mm.

These relations may be easily obtained with Trubyte teeth, which were formed with a knowledge of their necessity and advantages and have been carved to facilitate them. They can be obtained with other teeth only by extensive and tedious grinding, which, unavoidably, destroys the masticating efficiency of the teeth.

---

MESIAL INCLINATIONS OF ANTERIORS. If all the anteriors are set exactly vertical, the appearance of the denture will be too regular and mechanical.

If the upper laterals and cuspids are inclined mesially, the appearance of mechanical regularity and artificiality may be avoided. The teeth may also be rotated more or less on their vertical axes. The upper anteriors should be given the inclinations to the median line.

The roots of the upper centrals are parallel. The roots of the upper laterals and cuspids diverge from the median line, the laterals diverging slightly more than the cuspids.

The roots of the lower centrals and laterals are parallel, but those of the lower cuspids diverge strongly.

---

SETTING THE LOWER POSTERIORS. Cut away the rim of the lower wax trial plate from the median line on one side, almost to the heel. Set the lower first molar so that the buccal groove is opposite the mesial cusp of the upper first molar and wax it in place.

Move the articulator sideways in both directions. Note whether the upper buccal and lingual cusps pass through the occlusal grooves of the lower tooth, and whether the teeth separate or remain in articulation (which means "sliding contact").

The importance of an anatomical articulator here becomes apparent, since articulating relations can not be determined on a plain line articulator. We have so frequently mentioned the Gysi Simplex Articulator in this connection, that some dentists think Trubyte teeth can be articulated only on the Simplex. This is an error. Trubyte teeth can be arranged on any articulator and will do the best the articulator is capable of, but any teeth can be more accurately arranged on the Simplex than on any other except the Adaptable, because it more accurately reproduces the jaw movements.

When the lower first molars have been brought to correct working and balancing relations with the upper teeth, place and perfect the setting of the lower second bicuspid on the same side. Then set the lower first bicuspid.

Next set the lower first molar and second and first bicuspids on the opposite side and establish for them correct working and balancing relations when the articulator is moved first to one side and then to the other.

Then set and adjust the lower second molars on both sides.

If, while mastering the technic, much difficulty is experienced in securing proper balancing relations of the teeth, wax all the teeth firmly in position, mix carborundum powder with glycerine, place a little on the occlusal surfaces and make the upper and lower sets grind each other until a smooth and free movement with correct relations is obtained.

---

Labial Inclinations of Anteriors. Unless conditions in individual cases require setting the upper centrals to different inclinations, the incisal edges should be sloped outward. This setting is very effective with faces requiring nearly typal forms of teeth.

The neck of the upper lateral is pushed further in toward the ridge than that of the upper central. The cutting edge may well be slightly depressed, as compared with the edge of the central.

The upper cuspid sits more nearly vertical, as to labial slope, than either the central or lateral. That is, the neck is more prominent.

The lower centrals slope upward and labially outward more than the lower laterals or cuspids. When mechanical conditions permit this position, it is very effective in giving fine expression to the lower lip.

The necks of the lower cuspids are prominent in the degree required to give expression to the corners of the mouth and the cutting edges are inclined backward so that the labio-incisal margins occlude with the linguo-incisal margins of the upper cuspids.

---

WIDTHS OF LOWER ANTERIORS. Only the six lower anteriors remain to be set. These may be tried in place to see whether any changes in width have been made necessary by the manner of setting the upper anteriors. Trubyte upper and lower anteriors are carved to as nearly correct widths as possible, when set in the conventional manner, but any change in the form of the arch or in the manner of setting may necessitate slight changes in relative width.

If any narrowing of the lower anteriors is necessary, it can be best effected by grinding the distal sides of the lower cuspids and the mesial sides of the lower first bicuspids.

When the width of the lower anteriors, in relation to the uppers, has been determined and any necessary grinding accomplished, the lower incisors may be removed from the wax and the setting of the lower cuspids to right relations to the uppers perfected.

This relation is so important and knowledge concerning it is possessed by so few dentists, that the first task given students in the Laboratory of this Division is to grind upper and lower cuspids to right relations. Every one who has been here has felt that the mastering of this little step in technic has opened up a new understanding of tooth relations and of accomplishments, and several have said that the mastery of this one point alone was worth the work of the entire course.

It is not difficult and an explanation of the ends sought and the simple methods employed will make it possible to many who can not visit us.

